# Simultaneous Bilateral Quadriceps Tendon Rupture in a Patient with Diffuse Idiopathic Skeletal Hyperostosis after Minimal Trauma: Eight-Year Follow-Up

**DOI:** 10.1155/2018/5047138

**Published:** 2018-02-22

**Authors:** Sevan Sıvacıoğlu, Ahmet Salduz, Ufuk Öztürk, Serkan Bayram, Fevzi Birişik

**Affiliations:** ^1^Department of Orthopaedics and Traumatology, Surp Pirgiç Armenian Hospital, Istanbul, Turkey; ^2^Department of Orthopaedics and Traumatology, Faculty of Medicine, Istanbul University, Istanbul, Turkey; ^3^Department of Orthopaedics and Traumatology, Istanbul Research and Training Hospital, Istanbul, Turkey

## Abstract

**Introduction:**

The purpose of this report was to describe a very rare case of simultaneous bilateral quadriceps tendon rupture seen in a patient who was diagnosed as having diffuse idiopathic skeletal hyperostosis.

**Case Presentation:**

A man aged 64 years presented to the emergency department with bilateral quadriceps tendon rupture. Surgical repair was performed with suture anchors and a stainless steel cable. His legs were immobilized in casts for six weeks. After removal of the casts, physiotherapy was started. Four months after surgery, he was able to walk with 0°–120° range of motion and active extension. He was followed up for 8 years without rerupture or other complications.

**Conclusion:**

Bilateral rupture of the quadriceps tendon is a rare condition and generally related to metabolic disorders. Diffuse idiopathic skeletal hyperostosis is a metabolic disorder that causes bilateral quadriceps tendon rupture, and it accounted for the differential diagnosis of the underlying condition.

## 1. Introduction

In routine orthopedic practice, quadriceps tendon rupture is a common injury after major trauma in young patients. However, some metabolic disorders can cause spontaneous quadriceps tendon ruptures after minimal trauma. This extremely rare condition has been published in the literature with systemic diseases such as chronic renal failure, diabetes mellitus, rheumatoid arthritis, chronic tendinopathy, amyloidosis, and long-term use of systemic or local corticosteroid injections [1–3]. The purpose of this report was to describe a very rare case of simultaneous bilateral quadriceps tendon rupture in a patient who was diagnosed as having diffuse idiopathic skeletal hyperostosis (DISH).

## 2. Case

In 2008, a man aged 64 years was admitted to the emergency department with symptoms of pain and swelling in both knees and inability to walk. The history of the present condition was a fall from one step with minimal trauma.

**Figure 1:**
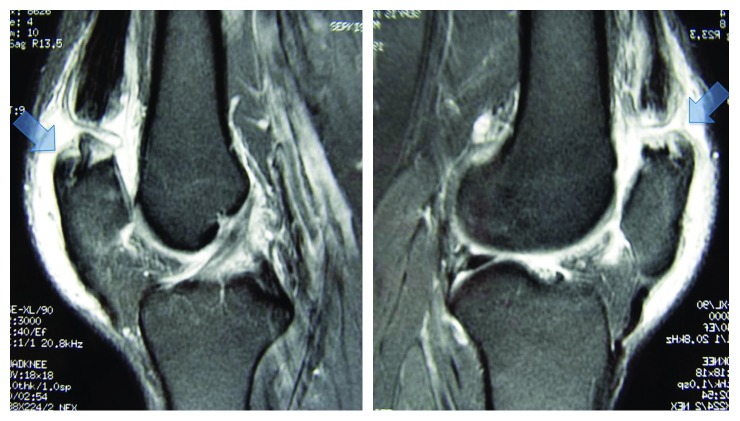
Sagittal T2-weighted MR image shows complete rupture of the quadriceps tendon with a gap between retracted quadriceps tendon and patella (right arrow). Left arrow shows the osteophyte formation at the upper end of the patella.

**Figure 2:**
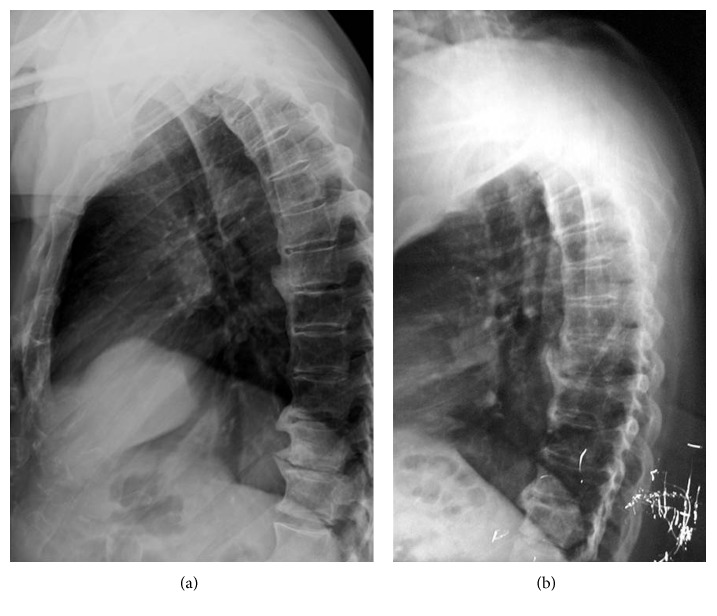
Kissing large osteophytes of the anterior longitudinal ligament is seen in the lateral view of the thoracolumbar spine (a). Kissing large osteophytes of the anterior longitudinal ligament is seen in the lateral view of the thoracal spine at the time of surgery in 2008 (b).

Physical examination revealed tenderness and a gap in both suprapatellar regions, no active motion in the extensor mechanism, and palpable defects in the quadriceps tendons with respect to attachment to the patella. There was only back pain in his medical history, which was treated with NSAIDs for ten years. There was no history of steroid use, fluoroquinolone antibiotic use, steroid injection, or chronic life-threatening disease. Initial X-rays were obtained. There was an osteophyte in the tibial tuberosity in the lateral left knee X-rays. Magnetic resonance imaging (MRI) was performed for further imaging. MRI showed the bilateral total rupture of the quadriceps tendons and showed the osteophyte in the tibial tuberosity in the left knee (Figure 1). A complete analysis revealed ossification and calcification of the anterior vertebral column in the spinal X-rays (Figure 2).

Laboratory tests were normal and showed a total leukocyte count (WBC) of 8600/mm^3^, an erythrocyte sedimentation rate (ESR) of 13 mm/h, and C-reactive protein (CRP) of 1.8 mg/l. Renal function was normal with creatinine (Cr) of 1.3 mg/dl and blood urea nitrogen (BUN) of 19 mg/dl. The patient's blood pressure was normal and blood sugar level was also normal (glucose: 83 mg/dl). HBA1C was not known. Cholesterol levels were normal (HDL: 57 mg/dl; LDL: 126 mg/dl). The patient has no metabolic syndrome. BMI was detected to be 24. The patient consulted the rheumatology department, and diffuse idiopathic skeletal hyperostosis was diagnosed when clinical and radiologic data were analyzed.

## 3. Surgery and Follow-Up

**Figure 3:**
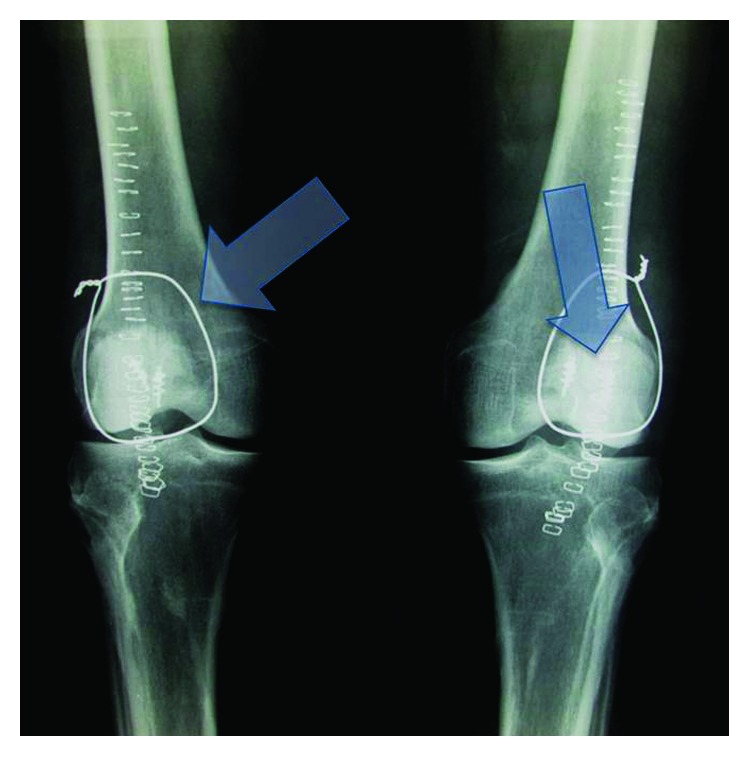
Early postoperative X-rays of the knees. Left arrow shows a cerclage wire, and right arrow shows the two 3.5 mm suture anchors.

The patient underwent surgery under spinal anesthesia 6 hours after the trauma. Both knees were prepared with routine cleaning and draping, and a tourniquet was applied. Longitudinal skin incisions were used for both knees. The distal ends of the quadriceps tendons were explored. The levels of the ruptures were very close to the patella. There were not enough tendons at the patellar side to repair end to end. The intraoperative findings were total rupture of the quadriceps tendons at the patella insertion. We observed in the calcification inside of the tendon especially in the distal side of the ruptured tendon perioperatively. It could be related to DISH. Two 3.5 mm suture anchors were placed at the proximal pole of the patella for both sides, and the distal part of the tendon was reattached using Krakow's method. Subsequently, a cerclage wire was passed around the patella and the proximal part of the quadriceps tendon to reinforce fixation (Figure 3).

The knees were immobilized in the knee casts for six weeks. The patient was able to walk after 3 weeks with cast. After cast removal, physiotherapy was started. The patient had 0°–120° pain-free range of motion in both knees 4 months after surgery with active extension. He could return to his job at the end of four months postoperatively.

**Figure 4:**
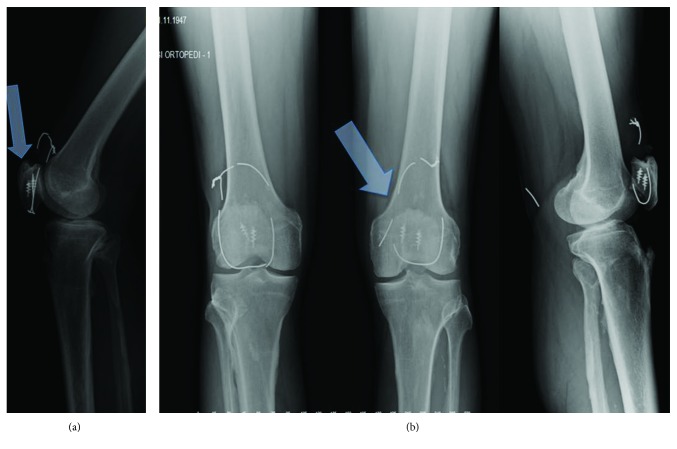
Bilateral knee X-rays after 8 years. Note the spur formations from the tibial tuberosity and patella (associated DISH) in the lateral view of the left knee ((b) right arrow). Bilateral cerclage wires have been broken ((a) left arrow).

**Figure 5:**
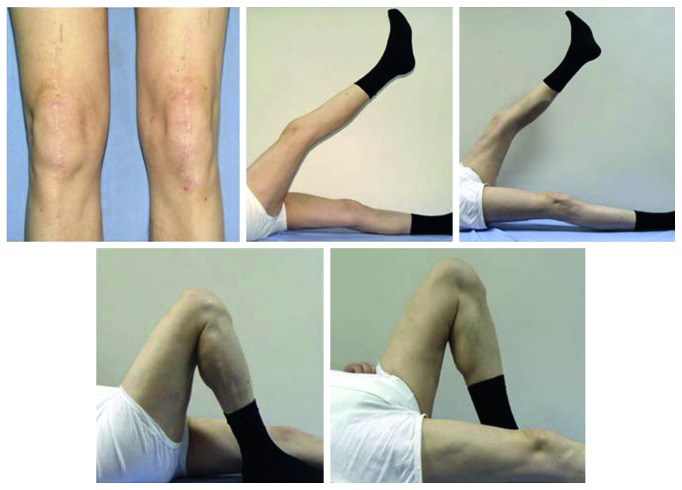
Clinical appearance of the patient's knees at eight years of follow-up. He has good functional results with full active motion in both knees.

There were no complications, wound infections, or joint limitations at the follow-up visits. At the last follow-up, eight years postoperatively, radiographs revealed broken wires, but the patient still had full range of motion in both knees with bilateral 15-degree extension lag (Figures 4 and 5). The Lysholm Knee Scoring Scale was applied to the patient, which showed 91 for the right knee and 95 for the left knee.

## 4. Discussion

DISH is characterized by ossification of the tendon, fascia, and ligaments. The vertebra is the most common site; pelvis, patellae, calcaneus, and olecranon can also be involved. The disease typically presents with ossification and the presence of flowing syndesmophytes in the vertebral column and without degeneration of discs. The etiology remains unknown. Obesity, type 2 diabetes, hypertension, and vitamin A toxicity drew attention for their association with the pathology of DISH. Involvement of thoracic, lumbar, and cervical vertebra disease is 97%, 90%, and 78%, respectively. Involvement of the disease in the whole spinal column is seen in 70% of patients. Dysphagia related with esophageal compression, pain, stiffness, and limitation of motion in the vertebra are common complications of this disease. It is three times more common in males, and most patients are aged over 50 years. The most commonly used diagnostic criteria were defined by Resnick and Niwayama [4]: (i) flowing calcifications and ossifications along the anterolateral aspect of at least 4 contiguous vertebral bodies, with or without osteophytes; (ii) preservation of intervertebral disk height in the involved areas and an absence of excessive disk disease; and (iii) the absence of bony ankylosis of facet joints and absence of sacroiliac erosion, sclerosis, or bony fusion, although narrowing and sclerosis of facet joints are acceptable [4]. Our patient has all the DISH criteria.

Quadriceps tendon rupture is a rare injury, but simultaneous bilateral quadriceps tendon rupture is exceptionally rare. These are generally reported as case presentations in the literature. Chronic renal failure, systemic lupus erythematosus (SLE), rheumatoid arthritis, diabetes mellitus, gout, and long-term corticosteroid use can cause quadriceps tendon rupture [1, 3, 5]. The mechanism of the injury is a generally strong contraction of the quadriceps muscle while the knee is in semiflexion and the ankle is in plantar flexion [6]. Patients with quadriceps tendon rupture are not able to walk after the injury and tend to hold their knee in an extension position to relieve pain [1, 6]. The physical examination generally consists of pain, inability of active extension of the knee, and a gap over the tendon. The suprapatellar gap is a pathognomonic sign for quadriceps tendon rupture [6]. In our case, there was no history of local or systemic corticosteroid use. Additionally, no joint or tendon pathologies had been reported before this injury. However, DISH was thought to be related to rupture of the quadriceps tendons in our patient, who had no other specific cause, based on the history and laboratory results. However, DISH is not limited to the spinal column and has often been reported to involve peripheral sites. Mader et al. described extra spinal diagnostic features of DISH as (i) involvement of joints usually unaffected by primary osteoarthritis; (ii) increased hypertrophic changes compared with primary osteoarthritis; (iii) prominent enthesopathies at various sites adjacent to peripheral joints; and (iv) calcification and ossification of entheses in sites other than joints [7]. As a characteristic feature of patients with DISH, spur formation can cause tendon problems and rupture. Ellanti et al. found that patellar bone spurs had significantly higher incidence in their series of quadriceps tendon rupture [8]. Hardy et al. analyzed the presence of patellar spurs in patients with patellar tendon rupture, patella fracture, and quadriceps tendon rupture. The authors showed that 23 of 29 (79%) patients had patella spurs in the quadriceps tendon rupture group [9].

The pathophysiologic mechanism of quadriceps tendon rupture in patients with DISH could be multifactorial. However, blood supply of the quadriceps tendon, microtrauma, collagen quality, and tendon calcifications have a role in quadriceps tendon rupture [10]. Polymorphism of the collagen gene is another rare risk factor for spontaneous ruptures.

Chronic renal failure is another cause of simultaneous bilateral quadriceps tendon rupture in the literature. Lee et al. [5] reported a case of simultaneous bilateral quadriceps tendon rupture in a patient aged 30 years with chronic renal failure and parathyroid adenoma. This patient was under hemodialysis treatment, and tendon rupture occurred spontaneously. In this situation, uremic toxins, beta 2-microglobulin, come together in tendons. The other way in patients with chronic renal failure is a calcium, phosphate, and parathyroid mechanism, which can cause tendon rupture. Glomerular filtration failure reduces serum calcium in this condition, parathyroid hormone elevates, and bone resorption begins [5]. It was found that duration of dialysis was a related mechanism of injury.

Some patients in the literature with arthritis or systemic lupus erythematosus had endothelial swelling and vascular mononuclear cell infiltration of the tendon area in microscopy studies. Patients with rheumatoid arthritis have higher levels of collagenase, and this can cause tendon rupture. In addition, it was found that chronic symptomatic tendinopathy was also related to quadriceps tendon rupture in older patients.

This is the first case report to demonstrate a possible relation between simultaneous bilateral quadriceps tendon rupture and diffuse idiopathic skeletal hyperostosis secondary to minimal trauma. This diagnosis should be taken into account in patients with quadriceps tendon rupture.
